# CRISPR/Cas9 System as a Valuable Genome Editing Tool for Wine Yeasts with Application to Decrease Urea Production

**DOI:** 10.3389/fmicb.2017.02194

**Published:** 2017-11-09

**Authors:** Ileana Vigentini, Marinella Gebbia, Alessandra Belotti, Roberto Foschino, Frederick P. Roth

**Affiliations:** ^1^Department of Food, Environmental and Nutritional Sciences, Università degli Studi di Milano, Milan, Italy; ^2^Donnelly Centre, University of Toronto, Toronto, ON, Canada; ^3^Departments of Molecular Genetics and Computer Science, University of Toronto, Toronto, ON, Canada; ^4^Lunenfeld-Tanenbaum Research Institute, Sinai Health System, Toronto, ON, Canada; ^5^Canadian Institute for Advanced Research, Toronto, ON, Canada

**Keywords:** CRISPR/Cas9 system, saccharomyces cerevisiae, wine, arginine degradation pathway, urea, ethyl carbamate

## Abstract

An extensive repertoire of molecular tools is available for genetic analysis in laboratory strains of *S. cerevisiae*. Although this has widely contributed to the interpretation of gene functionality within haploid laboratory isolates, the genetics of metabolism in commercially-relevant polyploid yeast strains is still poorly understood. Genetic engineering in industrial yeasts is undergoing major changes due to Clustered Regularly Interspaced Short Palindromic Repeats (CRISPR) and CRISPR-associated protein (Cas) engineering approaches. Here we apply the CRISPR/Cas9 system to two commercial “starter” strains of *S. cerevisiae* (EC1118, AWRI796), eliminating the *CAN1* arginine permease pathway to generate strains with reduced urea production (18.5 and 35.5% for EC1118 and AWRI796, respectively). In a wine-model environment based on two grape musts obtained from Chardonnay and Cabernet Sauvignon cultivars, both *S. cerevisiae* starter strains and *CAN1* mutants completed the must fermentation in 8–12 days. However, recombinant strains carrying the *can1* mutation failed to produce urea, suggesting that the genetic modification successfully impaired the arginine metabolism. In conclusion, the reduction of urea production in a wine-model environment confirms that the CRISPR/Cas9 system has been successfully established in *S. cerevisiae* wine yeasts.

## Introduction

While for laboratory strains of *Saccharomyces cerevisiae* several molecular methods have allowed extensive interpretation of gene functionality, industrial and wild yeast strains are still poorly studied; indeed, the genetic manipulation of latter yeasts can be time consuming because of they are usually recalcitrant to some molecular techniques and they are characterized by complex genomes (i.e., diploid and polyploid species). For this reason, the development of a rapid and efficient gene-targeting system based on the type II bacterial Clustered Regularly Interspaced Short Palindromic Repeats and CRISPR associated protein (CRISPR-Cas9) system is gaining attention in several industrial fields. Taking advantage of the high efficiency of homologous recombination (HR) in yeast, this system allows for double strand breaks and simultaneous gene editing of all copies of the target sequence (Gratz et al., 2013).

The CRISPR/Cas system, first discovered in *Escherichia coli*, is present in many eubacteria and archaea where it can provide resistance to bacteriophage or conjugative plasmids (Barrangou et al., 2007; Hryhorowicz et al., 2017). Foreign invading genetic material that is incorporated between CRISPR repeat sequences is transcribed and processed into CRISPR RNAs (crRNAs) that correspond to both foreign and CRISPR repeat DNA. The crRNAs hybridize with transactivating CRISPR RNAs (tracrRNAs) and the resulting crRNA/tracrRNA complex acts as a guide for the endonuclease Cas9, which cleaves invading nucleic acid sequences (Brouns, 2012; DiCarlo et al., 2013).

The main elements of the CRISPR/Cas9 system we used are a bacterial CRISPR-associated protein nuclease (Cas9), from *Streptococcus pyogenes*, and a short RNA guide. This latter element combines with Cas9 to target a specific DNA *locus* composed by 20 nucleotides and a NGG sequence, called protospacer adjacent motif (PAM), where the cleavage occurs in the nuclease domains RuvC and HNH (Mahfouz et al., 2014). The gRNA-Cas9 complex generates DSBs immediately before the PAM site on the target DNA (Ryan and Cate, 2014). Finally, the DSBs in the chromosomal DNA are repaired with knockouts/deletions or knock-ins/insertion by NHEJ (non-homologous end joining) and HR (homologous recombination) (Gratz et al., 2013).

Aside from the molecular advantage of producing quick genome changes by using a unique gene- editing approach, the CRISPR/Cas9 system has the potential to soon become the gold standard technique for the production of novel microorganisms suitable for the food industry. The system produces marker-free mutants and thus limits the environmental risk of using genetically modified microorganisms. Indeed, the system has been applied in many eukaryotic organisms (Komor et al., 2017) such as mammalian cell lines (Lee et al., 2015), insects (Gratz et al., 2013), and yeasts (DiCarlo et al., 2013; Ryan and Cate, 2014; Jakočiunas et al., 2015). It has also been applied in plants where genetic modifications introduced by genome editing can be indistinguishable from those introduced by conventional breeding, such that the plants might be classified different from traditional GMO (genetically modified organism) with environmental risk equivalent to that of conventionally-bred organisms (Bortesi and Fischer, 2015). Winemakers might also benefit application of this new approach to grapes and to yeasts, enabling better understanding of the connections between wine features and wine yeast genetics.

In wine, urea is a major precursor of ethyl carbamate (EC), the ethyl ester of carbamic acid (Weber and Sharypov, 2009). Urea is the metabolic intermediate in the arginine degradation pathway in *S. cerevisiae*, and accumulation of urea in wines generates EC via a reaction between ethanol and the carbamyl group of carbamic acid during wine storage. EC is found in fermented foods such as grape wine, sake, distillated spirits, bread, kimchi, yogurt (Lee, 2013). Stevens and Ough (1993) studied EC formation under different storage condition; the EC is usually found in significant amounts (0.01–0.025 mg/L) in wine and it increases dramatically at high temperature with a logarithmic increase when urea concentration decreases (Xue et al., 2015). EC is a carcinogenic compound in a number of mammalian species and it has been classified in March 2007 by the International Agency for Research on Cancer (IARC) in group 2A (probably carcinogenic to humans) from group 2B (possibly carcinogenic) (Lee, 2013). Several countries have limitations of the amount of EC in fermented food; for example, in Europe the determination of EC in foods is under study by EFSA. The determination of EC is difficult because of lack of physicochemical properties; gas chromatography with mass spectrometry and high- performance liquid chromatography with MS or FLD are methods for EC determination (Lu et al., 2015). Two methods have been developed for reducing EC levels in food; one is based on the monitoring of all steps of the production chain starting from the nitrogen fertilization of vineyards and the other one is based on the use of controlled temperature during storage (Weber and Sharypov, 2009). However, these two strategies are costly and often unworkable for small-scale wine producers.

To the best of our knowledge, there are no studies available that describe the use of the CRISPR/Cas9 approach in the wine field. In this study, we adopt a strategy to modify wine yeasts with the purpose of testing the robustness of this new molecular tool and offering a new engineering pipeline for further gene editing in specific metabolic pathways relevant for wine production. In this study, two commercial *S. cerevisiae* strains have been genetically engineered to eliminate the arginine permease encoded by *CAN1*, leading to strains with reduced urea production in laboratory and wine environments.

## Materials and methods

### Yeast strains and maintenance

*Escherichia coli* TOP10 served as plasmid host (Invitrogen, CA, USA). For plasmid-selective growth, the TOP10 strain was grown on LB [1% (w/v) Tryptone, 0.5% (w/v) Yeast Extract, 1% (w/v) NaCl] and 100 μg/L ampicillin. For solid media 2% (w/v) agar was included. Yeasts used in this work are two commercial wine strains of *S. cerevisiae*: AWRI796 (Maurivin, South Africa) and Lalvin EC1118 (Lallemand Inc, France). Cells were stored in YPD medium (10 g/L yeast extract, 20 g/L peptone, 20 g/L glucose, 5.5 pH) supplemented with 20% (v/v) glycerol at −80°C. Yeast pre-cultures were produced by inoculating glycerol stocks at 1% (v/v) in YPD broth at 30°C for 3 days.

### Drug sensitivity test by spot tests

*Saccharomyces cerevisiae* (AWRI796, EC1118) wine yeast strains were tested for their sensitivity to Geneticin (G418), Nourseothricin (Nat), Hygromycin B (Hyg), which is often used for the selection of transformed cells and Canavanine (Can), which is used, e.g., to select against diploid cells in the Synthetic Genetic Array method (Tong and Boone, 2006). Since possible interaction between drug and nitrogen source contained in a medium can occur (Cheng et al., 2000), the capability of strains to growth under the presence of G418, Nat, Hyg and Can was tested in two types of media both based on YNB without amino acids and ammonium sulfate (Sigma Aldrich, Germany) and 2% (w/v) glucose: (i) YNBA, contained 5 g/L of ammonium sulfate, and (ii) YNBG was added with 1 g/L of L-glutamic, as nitrogen sources. All drugs were prepared as stock solution in distilled water, sterilized by filtration on 0.22 μm filters and added to liquid or solid media after their sterilization in autoclave. Yeast pre-cultures were obtained in each medium after incubation in aerobic condition at 30°C for 3–5 days. After OD_600nm_ determination, yeast cultures were diluted to 0.1 OD_600nm_ in sterile water. Then, 1 mL of culture was centrifuged (10,000 g, 5 min) and pellet was washed once in 1 mL of sterile water. Five μL of cells were spotted on Petri dishes containing the corresponding solid media with 2% (w/v) agar and supplemented with different concentration of drug. In particular, cell sensitivity to antibiotics was assayed with: (i) G418 at 0.2, 0.4, 0.6, 0.8 g/mL, (ii) Nat at 0.1, 0.2, 0.3 g/mL, (iii) Hyg at 0.2, 0.4, 0.6, 0.8 g/mL, and (iv) Can at 0.1, 0.2, 0.4, 0.6 g/mL. Cellular growth was detected after 7 day at 30°C. Yeast growth in absence of any drug concentration was used as positive control. Spot tests to determine the drug sensitivity were carried out starting from two independent yeast cultures and in duplicate. The full capability of the investigated strain to grow under the tested condition was expressed by the sign “+”; the symbol “−“ was assigned when no isolated colonies were detectable; “±” indicated that a slight inhibition cell growth was observed for single isolated colonies; the sign “−“ meant that cells are sensitive to the tested concentration of drug.

### DNA manipulation

Plasmid DNA was prepared from *E. coli* (Sambrook et al., 1989). All restriction reactions were carried out according to manufacturer's instruction (New England Biolabs, MA, USA). PCRs were performed in a 25 μL reaction mixture composed of Phusion 2x master mix (Invitrogen, Carlsbad, CA, USA), 10 μM of forward and reverse primers and 80–100 ng DNA. The amplification cycle was an initial denaturation at 95°C for 5 min, 45 s at the annealing temperature (3°C lower than melting temperature) and 1.5 min at 72°C for the extension. Final extension took 10 min at 72°C. Amplicons were separated on 1% (w/v) agarose gel prepared in TBE buffer (0.09 M Tris, 0.09 M Boric acid, 2 mM EDTA) with 0.05 μg/L ethidium bromide and bands were UV visualized (Geldoc 1000 System, Bio-Rad Laboratories, California). Bands were extracted from gel, eluted in 50 μL of mQ water using the QIAquick Gel Extraction Kit (Qiagen, Hilden, Germany), and quantified by Qubit® dsDNA BR (Broad-Range) Assay Kits (Invitrogen). All ligation reactions were performed using Rapid DNA Ligation Kit (Thermo Fisher Scientific, MA, USA) according to operating instructions.

### Yeast transformations

The wine *S. cerevisiae* strains (AWRI796, EC1118) were subjected to two sequential transformations. Each transformation was completed in duplicate. For the first transformation, cells were treated with a lithium/acetate protocol according to the procedure described by Hill et al. (1991) and using 3 μg of transforming DNA. Recombinants were verified both by growth assay and PCR-based detection of the kanMX6 cassette. To measure growth, the wild type yeasts and three transformed clones of each *S. cerevisiae* strain were grown in duplicate in YNBG medium supplemented with diff erent concentrations of G418 (0, 200, 400, 800, 1,000, 1,200, 1,400, 1,800, and 2,000 μg/mL) using a Tecan Genios plate-reading spectrophotometer (Tecan, Germany). Specifically, fresh cell cultures in YPD medium (aerobic condition, 30°C, 24 h) were used to inoculate 100 μL of YNBG medium at 0.1 OD 600 nm in a 96-well plate. Cellular growth was monitored at 595 nm every 15 min for a period of 24 h. For the PCR confirmation, DNA was extracted by colony PCR protocol consisting in a treatment of 5 μL of one full size colony dissolved in 200 μL ddH_2_O with 20 μL of Zymolase lysis buffer [1 μL of 5 U/μL Zymolase (Zymo Research, CA, USA) + 99 μL phosphate buffer] at 37°C for 2/3 h. After a step at 95°C for 15 min and centrifugation at 2,000 rpm for 7 min (Hettich Zentrifugen, Mikro 200), 3–5 μL of supernatant were used for the amplification with primers GMX6_F and GMX6_R (Table 1).

**Table 1:** Materials used in the present study.

**Material**	**Description**	**References**
**STRAINS**		
*E. coli* TOP10	F^−^, *mcrA Δ(mrr-hsdRMS-mcrBC) φ80lacZΔM15 ΔlacX74 recA1 araD139 Δ(ara-leu)7697 galU galK rpsL (StrR) endA1 nupG*	Invitrogen (Carlsbad, CA, USA)
EC1118	*S. cerevisiae* Lalvin EC1118	Lallemand Inc, France
AWRI 796	*S. cerevisiae* AWRI 796 (Australian Wine Research Institute)	Maurivin, South Africa
ScEC1118*can1*	*S. cerevisiae* EC1118 Gly70stop *CAN1* (-GGC- → -TAG-)	This study
ScAWRI796*can1*	*S. cerevisiae* AWRI796 Gly70stop *CAN1* (-GGC- → -TAG-)	This study
**VECTORS**		
p414-TEF1p-Cas9-CYC1t	CEN6/ARSH4 origin, *TRP1*, TEF1p promoter, codon optimized Cas9 with C-terminal SV40 tag, Amp^R^	(DiCarlo et al., 2013); Addgene, USA
p426-SNR52p-gRNA.CAN1.Y-SUP4t	2μm ori, *URA3*, SNR52 promoter, gRNA CAN1.Y expression cassette, SUP terminator, Amp^R^	(DiCarlo et al., 2013); Addgene, USA
p414-G418-TEF1p-Cas9-CYC1t	CEN6/ARSH4 origin, *kan*MX6 cassette, TEF1p promoter, codon optimized Cas9 with C-terminal SV40 tag, Amp^R^	This study
p426-Nat-SNR52p-gRNA.*CAN1*.Y-SUP4t	2μm ori, *nat*MX6 cassette, SNR52 promoter, gRNA CAN1.Y expression cassette, SUP terminator, Amp^R^	This study
pFA6a	Extraction of the *kan*MX6 espression cassette (promoter and terminator TEF1)	Bahler et al., 1998
P4339	Extraction of the natMX espression cassette (promoter and terminator TEF1)	Tong and Boone, 2006
**PRIMERS**		
Nat_F	CGGCCGACATGGAGGCCCAGAATA (T_m_ = 78.4°C)	This study
Nat_R	CATATGCAGTATAGCGACCAGCATT (T_m_ = 65.7°C)	This study
GMX6_F	GGTACCCGACATGGAGGCCCAGAAT (T_m_ = 75.7°C)	This study
GMX6_R	TACGTACAGTATAGCGACCAGCATT (T_m_ = 59.7°C)	This study
CAN1_F	GACAAATTCAAAAGAAGACGCCGA(T_m_ = 66°C)	This study
CAN1_R	AAATATGATATAAGAGCGCCCACTG (T_m_ = 62°C)	This study
gRN_F	TGTAGTGCCCTCTTGGGCTA	This study
gRNA_R	TCGAGCGTCCCAAAACCTTC	This study
CAN1.can1.Y.90.NCOD	TTCACTTCAGCGTTCTGTACTTCTCCTTCATCTTCATCACCTATCTAATCCTCCATAGAGAACGTATCCTCGCCATTTACTCTCGTCGGG	DiCarlo et al., 2013
CAN1.can1.Y.90.COD	CCCGACGAGAGTAAATGGCGAGGATACGTTCTCTATGGAGGATTAGATAGGTGATGAAGATGAAGGAGAAGTACAGAACGCTGAAGTGAA	DiCarlo et al., 2013

The resulting G418-resistant transformed cells were exposed to a second transformation by electroporation following the method reported by DiCarlo et al. (2013) with few modifications. Briefly, cells were grown in YNBG liquid medium supplemented with 200 μg/mL G418 at 30°C up to stationary phase. Thus, 50 μL of pre-culture were inoculated in 100 mL of the same above medium and grown overnight. Cells have been collected by centrifugation (18,000 g for 10 min) between 0.7 and 1.5 OD_600nm_ and re-suspended in 25 mL of lithium/acetate buffer (0.1 M lithium acetate, 10 mM DDT, 10 mM Tris-HCl pH 7.5, 1 mM EDTA pH 8) at room temperature for 1 h. Yeast cells were then washed twice in 25 mL of cold ddH_2_O and once in 10 mL of 1 M cold sorbitol. After that, cells were pelleted 10,000 g for 15 min at 4°C and re-suspended in 100 μL of 1 M cold sorbitol. Each transformation treatment required 40 μL of competent cells and 10 μL of DNA containing 200 ng of vector expressing gRNA corresponding to the *CAN1* gene and the Nat resistance cassette, and 2 μg of donor dsDNA (Table 1). The transforming mix was kept on ice for 5 min before electroporation at 2.5 kV, 25 μF, 200Ω in 0.2 cm cuvettes (BioRad Micropulser, BioRad, CA, USA). One mL of 1 M cold sorbitol and YPD medium (1:1 ratio) was added immediately after the current application and the cell suspension was incubated at 30°C for 3–6 h in static condition. Recombinant clones were isolated first on selective plates of YNBG with 200 μg/mL G418 and 50 μg/mL Nat. Subsequently, cells were replicated on YNBG plus 100 μg/mL Can and transformants were verified by: (i) targeting the Nat cassette using the colony PCR protocol with Nat_F/Nat_R couple of primers (Table 1); (ii) amplifying (CAN1_F/CAN1_R primers) and sequencing the *CAN1* gene by an external provider (TCAG, Toronto, CA).

The transformation efficiency was calculated as the number of transformants generated per μg of supercoiled plasmid DNA (Hayama et al., 2002). All data are calculated by applying the algebraic average between the calculated transformation efficiencies of each independent treatment. The mutation efficiency was calculated as reported by Jakočiunas et al. (2015) by picking 5 clones resistant to canavanine and submitting them to Sanger sequencing, with the primer pair CAN1_F/CAN1_R, to confirm the mutation in the expected position of the target gene.

### Yeast fermentations

Fermentation trials were carried out in synthetic and natural grape musts. The composition of synthetic must grape was obtained from the OIV protocol (Directive 22/06/2012, Appendix I) with few modifications: 1.7 g/L YNB without amino acids and ammonium sulfate (Sigma-Aldrich, Germany), 115 g/L Glucose, 115 g/L Fructose, 5 g/L Tartaric acid, 3 g/L Malic acid, 0.2 g/L Citric acid, 2 g/L L-Arginine, pH 3.5. Two grape musts produced in the Franciacorta area (Brescia, Italy) in vintage 2016 were used in this study: a red grape must of Cabernet Sauvignon and a white grape must of Chardonnay. Sugar composition of grape musts was: Cabernet Sauvignon 123.8 g/L Glucose, 123.2 g/L Fructose and for Chardonnay 93 g/L Glucose, 99 g/L Fructose. The APA content of Cabernet Sauvignon was 47.35 mgN/L while Chardonnay contained 250 mg/L. To obtain a final content of APA as 250 mg/L, the Cabernet Sauvignon grape must was corrected with a 10 g/L Supervit solution (Enartis SC, Novara) containing ammonium sulfate, ammonium phosphate and thiamine. Two g/L of arginine were added before the cell inoculation to each grape must (Amerine and Ough, 1980).

Both synthetic and natural grape musts were used to carry out fermentation in flasks. Each strain was separately grown in YPD broth in aerobic condition at 25°C, overnight and then it was inoculated in the synthetic grape must and in the grape musts to obtain an initial cell concentration of about 2 × 10^6^ UFC/mL. Fermentations were performed in triplicate in 250 mL glass flasks containing 200 mL of medium. Fermentations in laboratory conditions were performed in aerobic conditions at 25°C while the ones in oenological conditions were run at 20 ± 2°C. In order to establish a limiting oxygen condition as happens in natural vinifications, flasks with grape must were capped with Muller's valves containing 12% (v/v) sulphuric acid. This enables escape of carbon dioxide and avoids oxygenation of the musts. While in synthetic must, yeast cell growth was monitored by OD at 600 nm. In natural grape musts it was also determined by CO_2_ loss through reduction of glass flask weight. At the end of the alcoholic fermentation, when no weight variation is detected for three consecutive days, wines were centrifuged at 18000 g for 5 min and supernatants were maintained at −20°C for further chemical analyses.

### Chemical determinations and statistical analysis

The content of L-arginine/urea/ammonia, ethanol and sugars (glucose/fructose) was determined by enzymatic kits (Megazyme, Ireland) following manufacturer instructions. All data are expressed by means of tree replications and standard deviation (±SD). Nitrogen content in natural grape musts was assayed by formol titration (Fracassetti and Tirelli, 2015). Urea yield values were subjected to the one-way ANOVA in order to infer the effect of strains (not-transformed and mutant yeasts); statistically significant differences between means were defined at *p*-value < 0.001.

## Results

The results presented here show the successful editing of the *CAN1* gene of two *S. cerevisiae* wine yeast strains using a CRISPR/Cas9 system that consists of three elements: two expression vectors carrying the Cas9 gene and the gRNA, respectively, and a donor dsDNA fragment. To select the most suitable selectable markers for plasmid construction, tests were conducted to assess the sensitivity of the yeast strains toward drugs generally used in genetic engineering trials. After transformation, mutant strains were tested for their capability of forming urea in synthetic and natural grape musts. Fermentations in synthetic must, containing arginine as sole nitrogen source, led to the quantification of the urea production in wild type vs. mutant strains in absence of the nitrogen catabolite repression. Whether experiments carried out in grape must with several nitrogen sources allowed to validate the actual contribution of the *CAN1* gene in the urea production in oenological conditions.

### Drug sensitivity of *S. cerevisiae* wine strains

*Saccharomyces cerevisiae* EC1118 and AWRI796 strains had not been previously analyzed for their resistance to common agents commonly used for selectable markers. Therefore, assessment of drug sensitivity was required to choose markers for vector maintenance and recombinant yeast strains. Due to a possible interaction between drug and nitrogen source contained in one medium (Cheng et al., 2000), the capability of strains to growth under the presence of G418, Nat, Hyg, and Can was tested in YNBA and YNBG. All spot tests were run in duplicate. All the tested yeast strains grew on both media without drug supplementation in 3 days at 30°C. After 7 days, differences in the ability of forming colonies were observed among the analyzed yeasts. Results showed that *S. cerevisiae* AWRI796 resulted more sensitive than the EC1118 strain to canavanine (Table 2). A higher drug sensitivity was detected when L-glutamic acid, rather than ammonium sulfate, was added to the medium (Table 2). In particular, this difference was observed in media with geneticin and hygromycin B. Indeed, in presence of these two drugs both strains grew up to a final concentration of 400 μg/mL when ammonium sulfate was added to the medium, while growth was already inhibited at 200 μg/ml in medium containing L-glutamic acid. Based on these data, all transformations and fermentation trials were carried out in YNBG medium (liquid or solid) and G418 and Nat cassettes were chosen as selectable markers.

**Table 2:** The first symbol on the left corresponds to the ability of growth in YNBA and the symbol on the right refers to the yeast growth in YNBG medium.

**Drug concentration (μg/mL)**	**G418**	**Nat**	**Hyg**	**Can**
0	+/+	+/+	+/+	+/+
50	nd/nd	−/−	nd/nd	nd/nd
100	nd/nd	−/−	nd/nd	AWRI796(−), EC1118 (±)/−
200	+/−	−/−	+/−	−/−
300	nd/nd	−/−	nd/nd	−/−
400	±/−	nd/nd	+/−	nd
600	−/−	nd/nd	−/−	nd
800	−/−	nd/nd	−/−	nd

*G418, geneticin; Nat, nourseothricin; Hyg, Hygromycin B; Can, Canavanine. Cellular growth is reported as: (+): full growth with no visible isolated colonies; (−): no cell growth; (±): countable isolated colonies; nd: not determined. Spot tests were repeated twice*.

### Construction of plasmids

**Figure 1:**
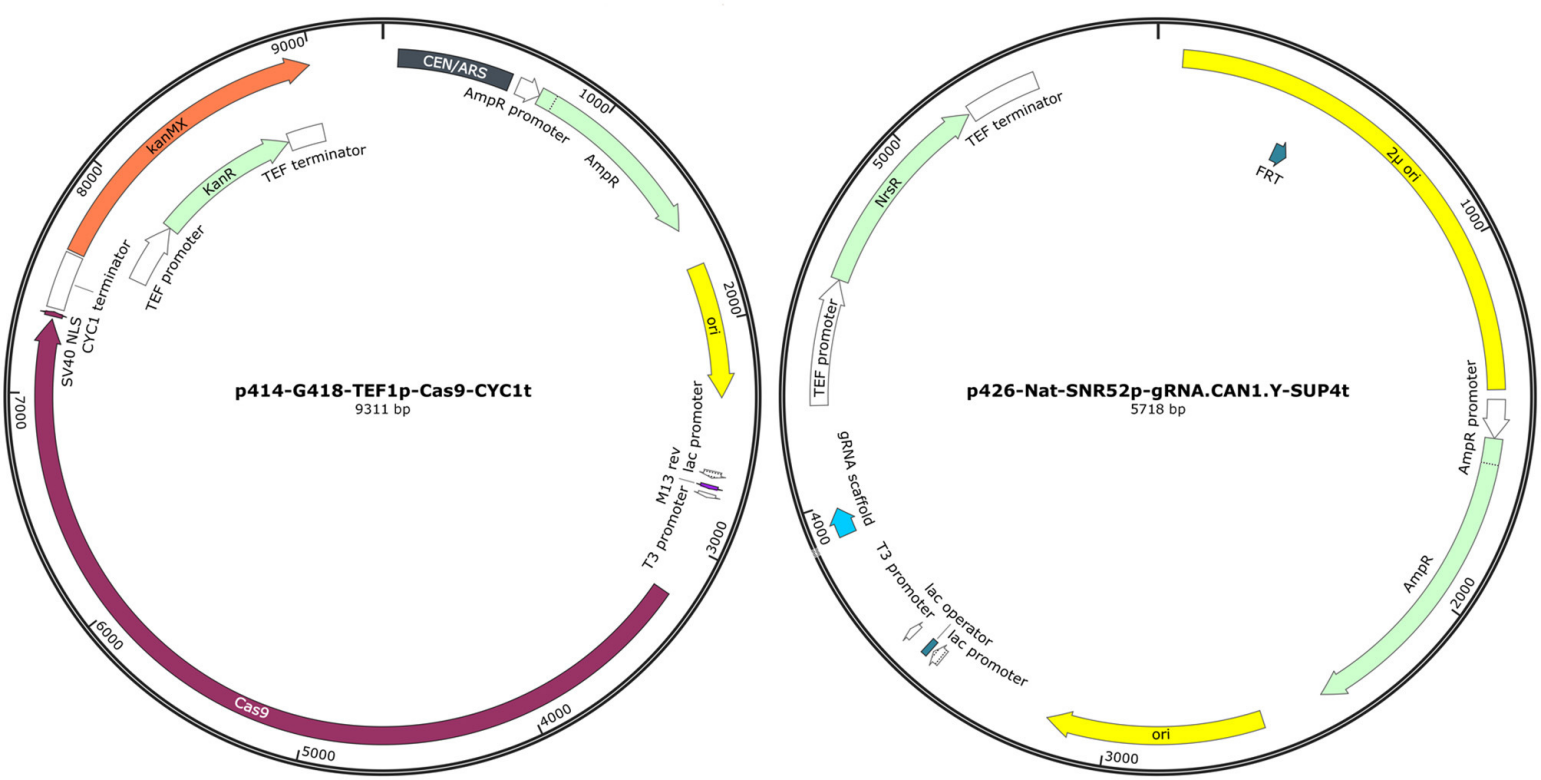
Representation of plasmid p414-G418-TEF1p-Cas9-CYC1t and p426-Nat-SNR52p-gRNA.CAN1.Y-SUP4t (SnapGene® Viewer 3.3.4).

All recombinant strains, plasmids and primer pairs used are listed in Table 1. The p414-G418-TEF1p-Cas9-CYC1t and p426-Nat-SNR52p-gRNA.CAN1.Y-SUP4t plasmids were obtained from p426-SNR52p-gRNA.CAN1.Y-SUP4t and p414-TEF1p-Cas9-CYC1t vectors, respectively. In vector p414-TEF1p-Cas9-CYC1t the *TRP1* gene was removed and replaced with the *kan*MX6 cassette (1365 bp), conferring resistance to G418. In the vector p426-SNR52p-gRNA.CAN1.Y-SUP4t the *URA3* gene was substituted with the *nat*MX cassette (1,126 bp) for the resistance to Nat. The cassette conferring Nat resistance was amplified from p4339 plasmid (Tong and Boone, 2006). The strategy used to change the selective markers of the original plasmids was similar for both new vectors. As a first step, *TRP1* cassette was excised from p414-TEF1p-Cas9-CYC1t using *Kpn*I/*Sma*BI enzymes and *URA3* was removed from p426-SNR52p-gRNA.CAN1.Y-SUP4t by digestion with *Eag*I/*Nde*I enzymes. Compatible ends at the 5′ and 3′ extremities of the *kan*MX6 and *nat*MX cassettes were generated by PCR amplification using primers GMX6_F/GMX6_R and Nat_F/Nat_R, respectively. Finally, the linearized plasmids and the corresponding resistance cassettes were ligated in order to generate p414-G418-TEF1p-Cas9-CYC1t (9,311 kb) and p426-Nat-SNR52p-gRNA.CAN1.Y-SUP4t (5,718 bp) plasmids (Figure 1).

### Transformation trials

**Figure 2:**
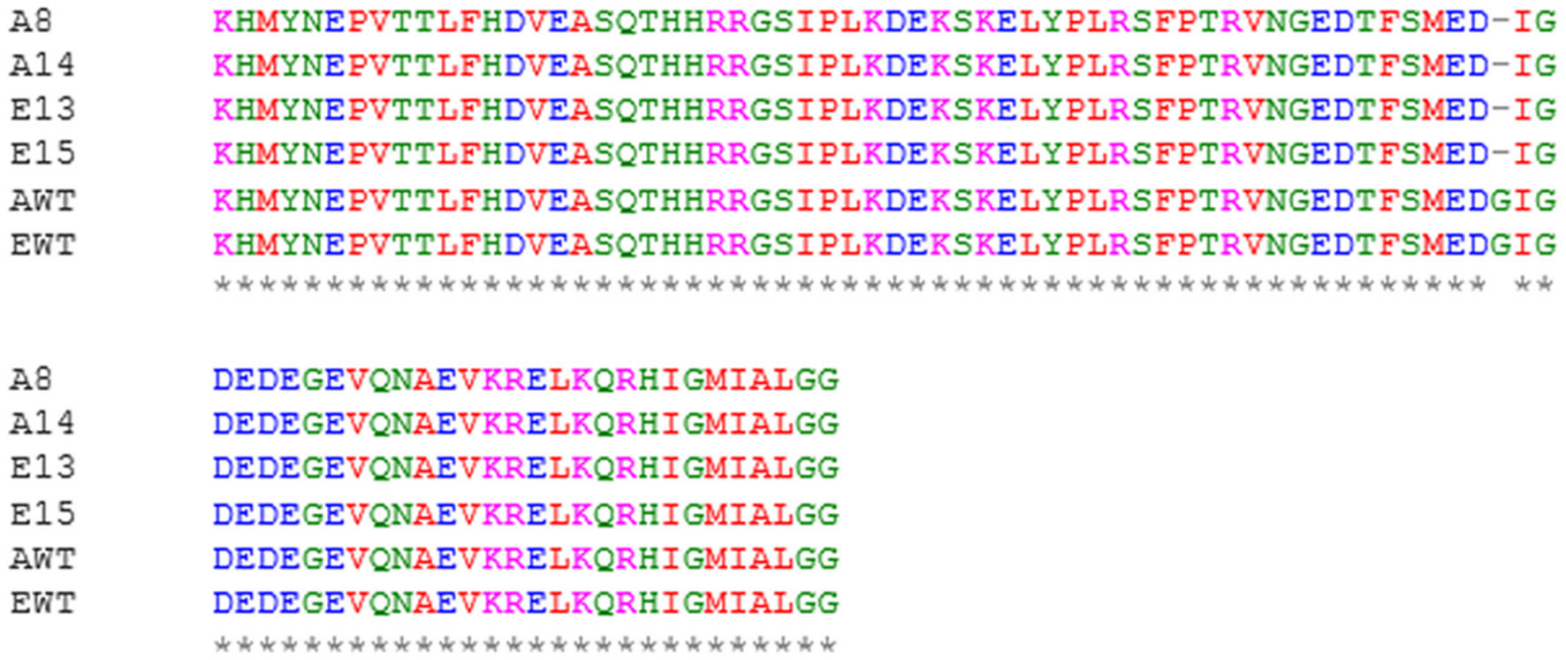
Example of the ClustalΩ multiple sequence alignment. Partial amino acid sequence of *CAN1* genes in wild type and recombinant strains: A8, *S. cerevisiae* AWRI796*can1* clone #8; A14, *S. cerevisiae* AWRI796*can1* clone #14; E13, *S. cerevisiae* EC1118*can1* clone #13; E15: *S. cerevisiae* EC1118*can1* clone #15; AWT, *S. cerevisiae* AWRI796, EWT, *S. cerevisiae* EC1118. A glycine amino amino residue (*G* = ggc) has been replaced by a STOP codon (− = tag) in position 70 from the methionine at the N-terminal of the protein.

During the first round of transformation, the plasmid p414-G418-TEF1p-Cas9-CYC1t (containing the *kan*MX6 marker for geneticin resistance) was transferred into cells using the lithium/acetate protocol applied to about 10^8^ cells per transformation reaction. While a transformation efficiency of about 222 ± 16 transformants per μg of DNA was calculated for *S. cerevisiae* AWRI796, a considerably lower value was obtained for *S. cerevisiae* EC1118 strain that showed a recovery of only 90 ± 6 transformants per μg of DNA. However, this difference was not confirmed by subsequent PCR assay for presence of the *kan*MX6 cassette; indeed, unlike what was observed for *S. cerevisiae* EC1118 where all the analyzed clones produced the expected fragment (1,365 bp), *S. cerevisiae* AWRI796 showed that only one to three clones had been correctly transformed. Finally, for each recombinant strain yielding positive PCR assay for the selectable marker, two isolates were further inoculated in liquid medium to assess G418 resistance. We found the *kan*MX6 cassette to confer resistance to the highest amount of antibiotic tested (2 mg/mL) (Figure 2). Interestingly, both clones of *S. cerevisiae* EC1118 showed the same growth fitness in YNBG, unlike *S. cerevisiae* AWRI796 which grew more poorly than wild type (data not shown).

**Figure 3:**
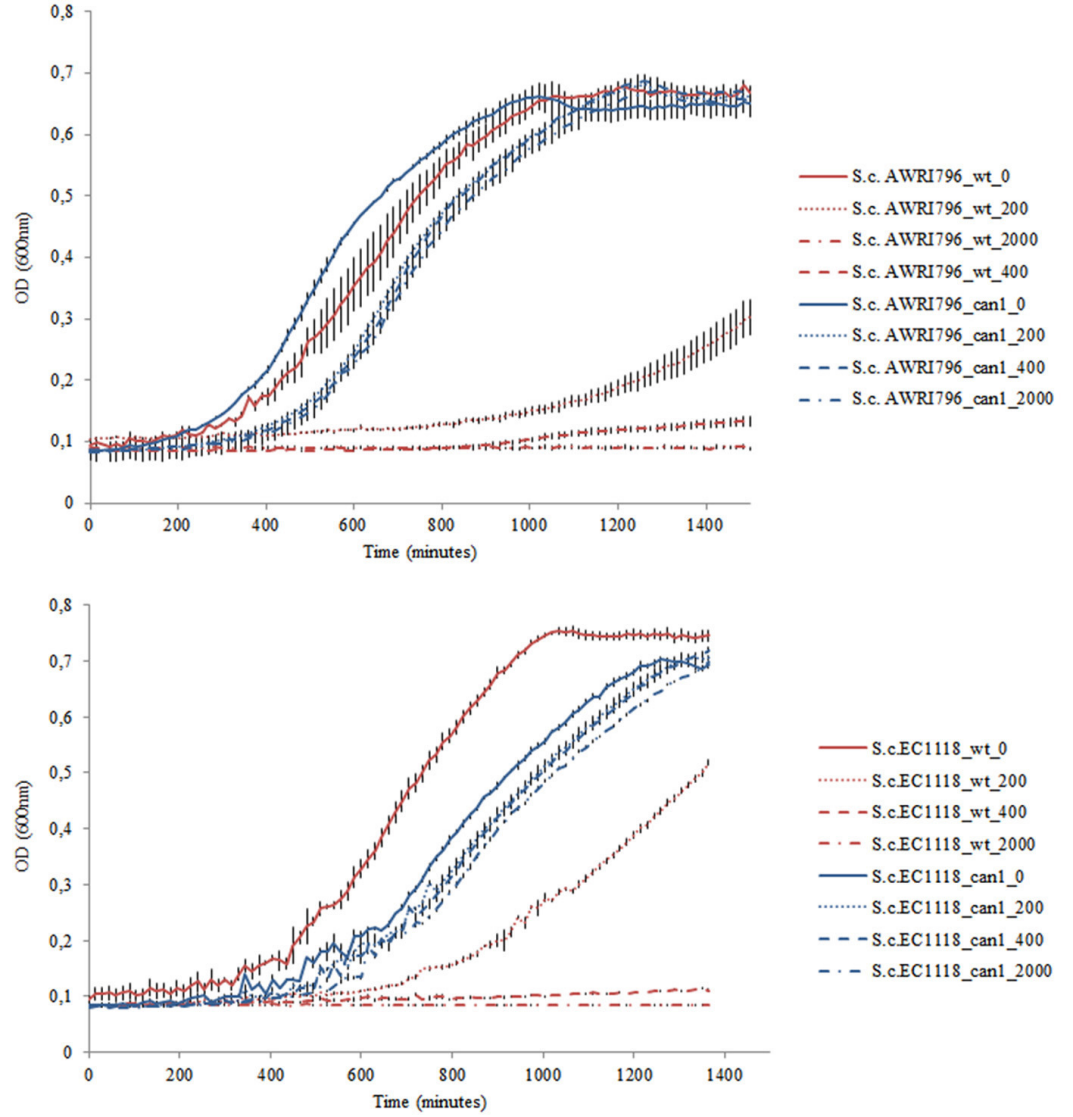
Example of the growth kinetics of *S. cerevisiae* wild type strains (AWRI796 and EC1118) and recombinant clones (clone #23 and clone#8 for AWRI796 and EC1118, respectively) inoculated in YNBG liquid medium in duplicate under different concentrations of G418 (0, 200, 400, and 2,000 μg/mL). Cellular proliferation was monitored for 24 h, 30°C using a Tecan microtitres reader (Tecan, Germany). The optical density was measured at 600 nm every 15 min up to stationary phase. Data are expressed by means of two replicates.

For the second transformation by electroporation, approximately 5 × 10^8^ cells containing the plasmid p414-G418-TEF1p-Cas9-CYC1t were co-transformed with the vector p426-Nat-SNR52p-gRNA.CAN1.Y-SUP4t (containing the *nat*MX marker for nourseothricin resistance) and the donor dsDNA. Selection occurred in YNBG agar medium supplemented with geneticin and nourseothricin. A transformation efficiency of (5.50 ± 3.25) × 10^3^ and (1.00 ± 0.17) × 10^4^ transformants for *S. cerevisiae* AWRI796 and EC1118 per μg of DNA was calculated, respectively. In this case, the high transformation efficiency could be linked to the time of cell recovery applied to electroporated cells (3 h at 30°C) before plating. Then, the recombinant cells underwent a second canavanine selection on YPD agar medium. Homologous recombination occurred with efficiency of 32 ± 2 and 22 ± 4 transformants per μg of DNA for *S. cerevisiae* AWRI796 and EC1118, respectively. The PCR amplification of the *nat*MX cassette confirmed the presence of the correct band at 1,126 bp from 10 selected clones per strain. Sequence analysis of the *CAN1* gene in the recombinant strains showed the presence of a stop codon at the expected position with a mutation efficiency of 100% of 5 isolates (Figure 3).

### Urea production from wild type and *can1* mutant strains

**Figure 4:**
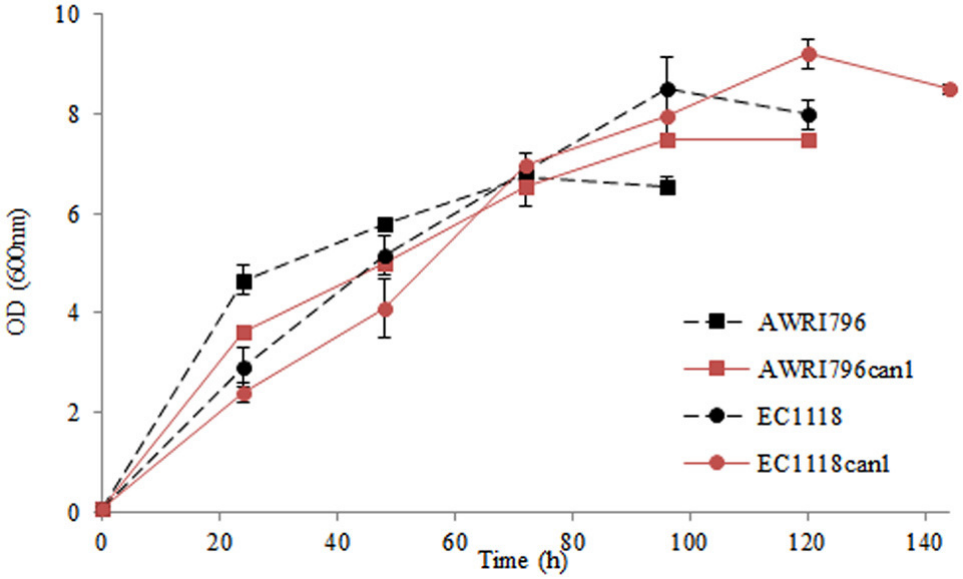
Kinetics of growth of wild types and recombinant strains in synthetic must. Data are expressed by means of three replicates and standard deviation (±SD).

In a chemically defined medium with arginine as sole nitrogen source, recombinant strains carrying the mutation in *CAN1* gene showed a statistically significant decrease in urea yield of 18.5–35.5% for *S. cerevisiae* AWRI796 and EC1118, respectively (Table 3). Indeed, a small amount of arginine was not consumed by mutant cells [0.1 g/L for AWRI796*can1* and 0.22 for EC1118*can1*, as compared with the AWRI796 and EC1118 wildtype strains that exhausted the available arginine (2 g/L)]. Interestingly, *S. cerevisiae* AWRI796*can1* showed a decrease in the specific growth rate in comparison to its wild type (0.07 vs. 0.09 h^−1^ μ_max_) (Figure 4).

**Table 3:** Urea yield and percentage of urea reduction in wild type and recombinant strains.

**Fermentation type**	**AWRI796**	**AWRI796*can1***	**EC1118**	**EC1118*can1***
**Synthetic must**				
Urea (g/L)	0.190 ± 0.008	0.150 ± 0.015	0.120 ± 0.005	0.080 ± 0.004
Biomass (g/L)	2.30 ± 0.20	2.35 ± 0.05	1.92 ± 0.18	2.10 ± 0.25
Urea yield	0.081^a^	0.066^b^	0.062^b^	0.040^c^
**Red must**				
Fermentative power (gCO2/250 mL)	22.1 ± 0.7	20.6 ± 0.6	22.0 ± 0.9	20.4 ± 0.3
Urea (g/L)	0.05 ± 0.002	n.d.	0.04 ± 0.002	n.d.
Biomass (g/L)	3.02 ± 0.08	2.23 ± 0.13	2.52 ± 0.075	2.38 ± 0.19
Urea yield	0.017^a^	0^b^	0.016^a^	0^b^
**White must**				
Fermentative power (gCO2/250 mL)	17.1 ± 0.5	16.1 ± 0.5	17.5 ± 0.5	17.0 ± 0.5
Urea (g/L)	0.020 ± 0.001	n.d.	0.01 ± 0.001	n.d.
Biomass (g/L)	2.55 ± 0.07	2.38 ± 0.13	2.50 ± 0.08	2.52 ± 0.02
Urea yield	0.008^a^	0^b^	0.004^a^	0^b^

*Yields were calculated at the maximum level of biomass (g/L). The standard error of enzymatic assays is calculated at 3%. The entry n.d = not detected (<0.13 mg/L, according to the detection limit of the enzymatic kit). The ANOVA was applied to urea yields calculated from fermentations performed in synthetic must and natural musts (red and white), respectively. Mean values, on the same line, showing statistically significant differences (p-value < 0.001) are superscripted with different letters*.

**Figure 5:**
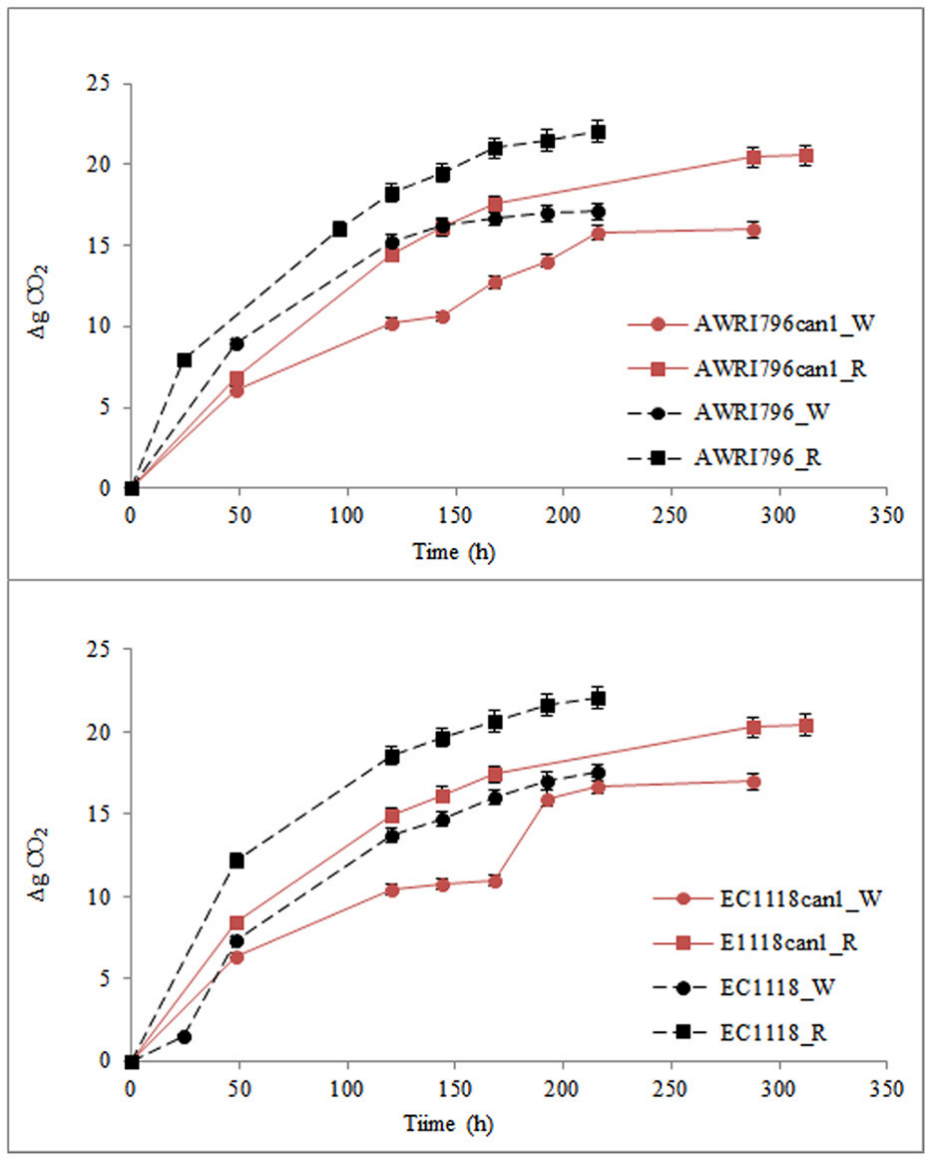
Production of CO_2_ by *S. cerevisiae* wild type (AWRI796 and EC1118) and recombinant strains (AWRI796*can1* and EC1118*can1*) in oenological conditions. R, Cabernet Sauvignon must; W, Chardonnay must. Data are expressed by means of three replicates and standard deviation (±SD).

Must fermentations were carried out inoculating *S. cerevisiae* AWRI796, AWRI796*can1*, EC1118 and EC1118*can1* strains in two grape musts obtained from Chardonnay (white) and Cabernet Sauvignon (red) cultivars. The two wild type strains completed the must fermentation in 8 days in both musts while the two mutants ended their growth at the 12th and at the 9th day in red in white musts, respectively (Figure 5). The biomass production ranged from 2.23 to 3.02 g/L as an average for all the tested strains, both wild and transformed; starting from the same amount of sugars as for in synthetic must, the presence of a complete pool of amino acids improved the cellular growth (Table 3).

In both musts, although the wild type strain of *S. cerevisiae* AWRI796 and EC1118 showed a better fermentative power in comparison to the mutant strains (total grams of CO_2_ produced/volume of fermentation) (Figure 5), the ANOVA highlighted that statistically significant differences were not found among strains (Table 3). Comparing the two transformed yeasts in terms of oenological traits, *S. cerevisiae* AWRI*can1* and EC1118*can1* were most performant in red rather than white must with a fermentative vigor (g CO_2_ produced in 48 h) of about 6.9 and 8.4 g, respectively. Although the mutant strains are able to finish the fermentation in both white and red must, they showed a delay of about 4 days; in terms of sustainability, this behavior should be better investigated if strains are used in a real oenological condition.

Finally, both *can1* mutants failed to produce urea (Table 3) and a lower consumption of arginine was detected in fermentations carried out with the *can1* mutant strains in comparison to those with wild types (1.3 vs. 1.7 g/L).

## Discussion

Selected yeast starter strains are widely used because they possess very good fermentative and oenological capabilities, contributing to the standardization of fermentation process, wine quality and safety. However, due to their polyploid nature, these strains are still poorly characterized from a genetic point of view. Here we outline a strategy to modify wine yeasts with the CRISPR/Cas9 system, an efficient, cheap and easy-to-use tool for genome editing that allows the simultaneous modification of all the alleles of a target gene. To prove the robustness of the CRISPR/Cas9 system in wine strains of *S. cerevisiae* and to provide a gene-editing pipeline suitable for metabolic pathways relevant in wine production, two commercial strains of *S. cerevisiae* (EC1118, AWRI796) have been genetically engineered in *CAN1* genes to generate strains with a reduced urea production. The *CAN1* gene, which encodes plasma membrane arginine permease, was selected as a model gene for its dual significance: (i) it allows the system validation by a negative selection of the transformed clones using canavanine and (ii) it represents the first enzyme of the arginine degradation pathway that is involved in the production of urea, the main precursor of ethyl carbamate (EC), a toxic compound (Ough et al., 1988).

Urea can be released by wine yeasts as the metabolic intermediate from arginine breakage (Vincenzini et al., 2009). According to this path, arginine is transported into the cell through specific and/or general amino acid permeases (encoded by *CAN1* and *GAP* genes, respectively) and is cleaved by arginase (*CAR1* gene) into ornithine and urea. Urea can then be excreted through Dur4p, a passive urea permease, or transformed by Dur1p/Dur2p, two urea amidolyases, in ammonium and CO_2_. Urea can undergo to a spontaneous, non-enzymatic, reaction with ethanol forming EC, which is known to be genotoxic and carcinogenic in a number of mammalian species and which affects several fermented food products. The development of techniques to prevent and/or reduce its content in wine represents an important goal in wine industry. Genetically modified yeasts in the genes of the arginine degradation pathway have been already been obtained for sake and sherry wine production (Coulon et al., 2006; Chiva et al., 2009; Dahabieh et al., 2010; Wu et al., 2014; Zhao et al., 2014). However, no study has investigated the role of *CAN1* gene in the production of urea in any fermentable source yielding human-consumed products.

By exploiting the CRISPR/Cas9 system, in the present work we generated *can1* mutants in *S. cerevisiae* wine strains in order to investigate the urea production in oenological conditions. Prior deciding how to construct useful vectors, yeasts were analyzed for their sensitivity to drugs commonly used in biotechnological studies. For all the analyzed compounds, we observed different levels of inhibition in the growth due mainly to the nitrogen source present in the media as shown by Cheng et al. (2000) and, only in the case of canavanine in rich medium, on the strain. We also assessed the efficiency of each step in our sequential transformation protocol. Low transformation efficiency was observed for the vector expressing Cas9p. While a value of about 3–5 × 10^4^ transformants/μg of plasmid DNA was expected (Hill et al., 1991), an efficiency of two order of magnitude less was calculated. Two possible hypotheses can be formulated; first, the plasmid size was too large (9,311 bp for p414-G418-TEF1p-Cas9-CYC1t) and reduced DNA uptake and/or Cas9p expression yielded toxicity leading to cell death (Ryan and Cate, 2014). By contrast, the second transformation, mediated by electroporation, yielded an efficiency similar to that reported in literature (Gysler et al., 1990; Pribylova and Sychrova, 2003). Finally, the *CAN1* gene was successfully modified with a 100% mutation frequency for both wine strains as shown by DiCarlo et al. (2013) for a lab strain of *S. cerevisiae*.

The resulting phenotypes of the *can1* mutants were evaluated in a wine-model environment using laboratory and oenological conditions. In a synthetic must, recombinant strains carrying the mutation in *CAN1* gene show a decrease in urea production between 18.5 and 36.5%. This result can be due to presence of the *GAP1* gene, the general acid permease gene that transports arginine into the cell (Chiva et al., 2009). In fact, the *GAP1* deletion could further reduce urea production (Wu et al., 2014) but it might also further reduce specific growth rate due to a reduced intake of arginine and other amino acids into the cells. In this study, possibly because of the metabolic modification of *CAN1* gene, a variation of the specific growth rate was observed in *S. cerevisiae* AWRI796*can1* in comparison to its wild type (0.07 vs. 0.09 h^−1^ μ_max_). Can1p inactivation may have an effect on the specific growth rate due to a reduced arginine uptake; however, further physiological experiments should be carried out to verify this metabolic behavior.

Since arginine is the most common organic nitrogenous compound in grape juice, the growth rate and the biomass production in *can1* mutant strains could be more affected than the wild type. In a wine-model environment, consisting of two grape musts obtained from Chardonnay and Cabernet Sauvignon cultivars, all the analyzed yeasts completed the must fermentation. The most important result is that recombinant strains, carrying only a mutation in the *CAN1* gene, show a complete reduction of urea in both musts. Of course, this achievement has yet to be confirmed in other musts and under actual winemaking conditions. Yeast can sense the numerous available nitrogen sources in a medium and “tune” nitrogen catabolite repression toward optimal utilization of nitrogen. In a grape must, the presence of ammonia, a yeast could down-regulate a pathway necessary to import arginine or other amino acids. Therefore, rather than changing other enzymes of the arginine degradation pathway with possible consequences on the yeast fitness, the sole mutation in *CAN1* could be enough to reduce the urea production.

In conclusion, this study demonstrates that CRISPR/Cas9 system can be successfully established in *S. cerevisiae* wine yeasts, and the editing of the *CAN1* gene thereby yields a reduction of urea production.

## Author contributions

IV contributed to the design of the work, to the construction of plasmids and to perform the spot tests for drug sensitivity. IV contributed also contributed to the acquisition, the analysis, and the interpretation of data for the work, to draft the work and revising it and ensured that questions related to the accuracy or integrity of any part of the work were appropriately investigated and resolved; MG contributed to the design of the work and the implementation of the CRISPR/Cas9 system, to the interpretation of data for the work, to draft the work and revising it; AB contributed to the fermentation growth of yeast strains; RF contributed to the interpretation of data for the work and to draft the work; FR contributed to draft the work and revising it for important intellectual content and to ensure that questions related to the accuracy or integrity of any part of the work were appropriately investigated and resolved.

### Conflict of interest statement

The authors declare that the research was conducted in the absence of any commercial or financial relationships that could be construed as a potential conflict of interest.
